# Long-Term Durability of CFRP Strips Used in Infrastructure Rehabilitation

**DOI:** 10.3390/polym17131886

**Published:** 2025-07-07

**Authors:** Karunya Kanagavel, Vistasp M. Karbhari

**Affiliations:** 1 Department of Civil Engineering, University of Texas Arlington, Arlington, TX 76019, USA; 2 Department of Mechanical and Aerospace Engineering, University of Texas Arlington, Arlington, TX 76019, USA

**Keywords:** composite materials, carbon, epoxy, pultruded, water, saltwater, alkaline, tension, durability

## Abstract

Prefabricated unidirectional carbon fiber reinforced polymer (CFRP) composite strips are extensively used as a means of infrastructure rehabilitation through adhesive bonding to the external surface of structural concrete elements. Most data to date are from laboratory tests ranging from a few months to 1–2 years providing an insufficient dataset for prediction of long-term durability. This investigation focuses on the assessment of the response of three different prefabricated CFRP systems exposed to water, seawater, and alkaline solutions for 5 years of immersion in deionized water conducted at three temperatures of 23, 37.8 and 60 °C, all well below the glass transition temperature levels. Overall response is characterized through tensile and short beam shear (SBS) testing at periodic intervals. It is noted that while the three systems are similar, with the dominant mechanisms of deterioration being related to matrix plasticization followed by fiber–matrix debonding with levels of matrix and interface deterioration being accelerated at elevated temperatures, their baseline characteristics and distributions are different emphasizing the need for greater standardization. While tensile modulus does not degrade appreciably over the 5-year period of exposure with final levels of deterioration being between 7.3 and 11.9%, both tensile strength and SBS strength degrade substantially with increasing levels based on temperature and time of immersion. Levels of tensile strength retention can be as low as 61.8–66.6% when immersed in deionized water at 60 °C, those for SBS strength can be 38.4–48.7% at the same immersion condition for the three FRP systems. Differences due to solution type are wider in the short-term and start approaching asymptotic levels within FRP systems at longer periods of exposure. The very high levels of deterioration in SBS strength indicate the breakdown of the materials at the fiber–matrix bond and interfacial levels. It is shown that the level of deterioration exceeds that presumed through design thresholds set by specific codes/standards and that new safety factors are warranted in addition to expanding the set of characteristics studied to include SBS or similar interface-level tests. Alkali solutions are also shown to have the highest deteriorative effects with deionized water having the least. Simple equations are developed to enable extrapolation of test data to predict long term durability and to develop design thresholds based on expectations of service life with an environmental factor of between 0.56 and 0.69 for a 50-year expected service life.

## 1. Introduction

Fiber reinforced polymer (FRP) composites provide significant opportunities, and advantages, for use in civil infrastructure and the overall topic has been extensively reviewed over the years with a number of recent publications assessing both the state-of-the-art and challenges [1,2,3,4,5,6,7,8], and hence reviews of the topic will not be repeated herein. Among the various applications of FRP composites, its use as externally bonded, or near-surface mounted, reinforcement to rehabilitate/strengthen deteriorating and/or understrength structural elements derives specifically from its lightweight, high specific strength and stiffness, ease of application in the field, and potentially long service life. Both prefabricated elements (strips and rods) that are bonded onto the concrete substrate, or placed in grooves cut into the surface, using appropriate adhesives, and fabric based composites that are simultaneously laid up and bonded using the wet layup process with the resin serving as the bond layer, have been shown to be effective [9,10,11,12]. The characterization specifications for the materials as well as the development and application of codes, standards, and specifications for the use of FRP composites in civil infrastructure applications is discussed in [13,14,15,16,17].

Notwithstanding the successful application of such systems, there is still a lack of standardization among available products both due to the variation inherent in the process used to fabricate the composites, such as the wet layup process which is largely manual, or due to variation in available forms as in prefabricated strips/bars. This leads to variability in properties and mechanical characteristics which create a level of uncertainty especially in the development of the basis for design thresholds. Studies on factors affecting properties and the degree of uncertainty in these have been conducted through a number of studies [18,19,20,21,22,23,24,25] including assessing the best-fit statistical distributions. However, further work is still required to ensure appropriate development of a unified standard and set of specifications [13,18].

In addition, there are still concerns related to the determination of long-term durability and the prediction of service-life [5,6,8]. For the most part, external strengthening is effected through the use of carbon fibers as the reinforcing phase in an epoxy-based composite. While these fibers are inert to most environmental conditions likely to be faced by civil infrastructure components and systems, the resin and the fiber–matrix interface are still susceptible to deterioration as a result of exposure to humidity, solution, heat, and UV, among others. In particular, exposure to humidity, or solution, results in moisture uptake which can result in a series of mechanisms that degrade the composites and could affect its overall integrity, including through plasticization, hydrolysis, and saponification. The lack of reliable sets of long-term durability studies and a limited understanding of the long-term performance of non-aerospace composites have been noted to be significant barriers to the greater use of these materials in civil infrastructure [26]. This is especially true when considering traditional expectations of service lives of 50 years or greater [27]. This has led to increased attention to the determination of long-term durability through extended term laboratory tests under controlled conditions of exposure, attempts to correlate results from tests conducted in outdoor environments to those in laboratories, and through testing of limited specimens extracted from field applications.

While there have been a significant number of successful field implementations of externally bonded composites to strengthen deteriorating elements, and/or to increase service-life, very few have been accompanied by extensive test programs that assess changes in materials characteristics over time. Reay and Pantelides tested specimens from a bridge in Utah on Interstate 80 at intervals of up to 30 months of field exposure reporting decreases in tensile strength between 3.6 and 16.8% of the baseline [28]. Allen and Atadero [29] tested specimens from a field site and found that the tensile strength of externally bonded FRP which was determined using the Colorado DOT’s requirement of Mean–3 (standard deviation) was 288 MPa, significantly lower than the manufacturer provided specification of 876 MPa and the design threshold of 745 MPa. However, it was not clear as to whether the decrease was due to degradation over time or due to poor installation procedures. Milev and Tartar [9] tested specimens peeled from a rehabilitated girder after 26 years of service and noted tensile strengths 37% less than that of data specifications used in the rehabilitation with longitudinal matrix splitting and microcracking. They determined that the value of the environment reduction factor, C_E_, to attain this level should be 0.66. Alsuhaibani et al. [10] tested specimens from a 12-year retrofitted bridge and reported appreciable degradation in tensile strength of 21% although the modulus was noted to decrease by only 3%. They concluded that the ACI-440 [30] specified environmental reduction factor of 0.85 overestimates levels and could result in an unsafe design. They suggested a reduced level of 0.75 and emphasized that the loss in modulus could be as high as 25% and thus needed to be considered as well. While limited, the cases emphasize the differences in results obtained in the laboratory and field and the critical need for better prior planning for field tests through the life of the rehabilitated structure.

While results from field investigations provide the most realistic assessment of the response of composites to exposures faced by civil infrastructure components in the field, these are both scarce and difficult to conduct in a structured manner due to the need for good records, a significant set of test samples available over extended periods of time extracted from the strengthened component system without causing undue deterioration, and the ability to correlate effects to primary components of exposure conditions which can change daily. Due to these difficulties, laboratory studies are more extensive and summaries can be found in [5,6,14,26,31,32,33,34] amongst others. Rather than repeat general results and trends already covered in the literature, a few recent results pertinent to composites used in external strengthening will be highlighted. Ortiz et al. [14] provide a fairly extensive review of the durability of FRP rods and cite a paper by Benmokrane et al. [34] as the source for changing the previous ACI value of C_E_ from 0.7 or 0.8 [35] to 0.85 based on 361 accelerated aging tests of unstressed bars. The same factor is used for the design of FRP used for external strengthening. However, it must be noted that the FRP strips used in externally bonded applications have a significantly lower thickness than the diameter of FRP bars resulting in far faster transport of moisture through the thickness and hence earlier initiation of deterioration at the levels of the fiber–matrix interface and in the matrix itself. In addition, these plates/strips do not have a substantial resin rich coating as found on bars, again resulting in faster effects of the exposure resulting in greater drops in mechanical and interfacial characteristics. This effect is highlighted by Liu et al. [36] who reported rapid degradation in short beam shear (SBS) strength in 7 mm diameter pultruded rods where water uptake resulted in the breaking of van der Waal’s and hydrogen bonds resulting in degradation of interfaces with levels of 15.5%, 22.3%, and 29% degradation of SBS strength due to immersion in water at 40°, 60°, and 80 °C, respectively. Based on their model, a level of 90% retention was attained at the relatively short period of just under 2 years (611 days) at 21.8° C. Cabral-Fonseca et al. [37] studied the flexural strength retention of three types of pultruded carbon fiber reinforced polymer (CFRP) strips and reported flexural strength retention levels of 68%, 68%, and 89% in these systems when immersed in water at 60° in comparison to levels of 53%, 69%, and 99% for the same systems in salt water. In addition, immersion in alkaline solution resulted in two systems having degraded to such an extent after 18 months that the test could not even be conducted, emphasizing the accelerated, and high levels of, degradation of the fiber–matrix interface. Dushimimana et al. [38] attempted to establish relationships between accelerated, and natural, aging of the same materials but were unable to reach a conclusion and attributed this to only having data pertaining to short periods of exposure. In a continuation of that work Dushimimana et al. [39] reported that one set of strips immersed in room temperature water showed an increase in strength and modulus after four years while a second set showed a 4.2% decrease in strength and 14.7% decrease in modulus with decreases between 1.4% and 6.3% in strength from outdoor exposure over a range of conditions based on location and between 5.3% and 13.7% decrease in modulus over four years. Of special significance is that none of the field exposures showed increases in strength and modulus over time unlike the laboratory set. Zhou et al. [40] reported on the durability of carbon/epoxy fabricated using a hot compression molding process and reported losses of 5%, 35.2%, and 26.1% in flexural strength when immersed in water at 25°, 60°, and 80 °C, respectively for 30 days in comparison to unexposed specimens with degradation continuing to increase with time. Li et al. [41] reported significant degradation in tensile strength and interfacial shear strength due to immersion in sea water for 100 days due to disruption of the fiber–matrix interface and weakening of the matrix due to plasticization. Drops of 11.6% and 16.6% were noted in tensile strength due to immersion in seawater at 23° C and 60 °C, respectively, with significantly greater drops of 27.9% and 35.8% in interfacial shear strength under the same conditions, while the drop in tensile modulus was only 3.4 percent in room temperature sea water but 16.5% at 60 °C after immersion for 100 days. Bone at al [42] reported a drop of 20% in flexural strength and 10% in flexural modulus as a result of 56 days immersion in water at 60° C. These results, among others, reiterate not only that mechanical properties can drop significantly over periods of time, but that short-term exposure does not always provide indicators representative of longer-term response, and that interfacial effects can be severe. This emphasizes both the need for development of larger, well-characterized, datasets on the basis of which more accurate factors for environmental degradation can be determined, and the need to also focus on properties such as SBS- or flexural-strength in addition to the more commonly measured tensile characteristics in which results of deterioration due to exposure and aging over longer periods of time can be obscured.

The current investigation focuses on the assessment of durability of three different, commercially available, prefabricated CFRP strips used in the externally bonded strengthening of structural concrete through laboratory tests conducted for a period of 5 years, thereby providing a significantly longer period of assessment of mechanisms accruing from hydrothermal exposure and thus a better basis for the prediction of long-term response in tension and short-beam-shear. The investigation is part of a larger study of the long-term durability of FRP materials used in civil infrastructure applications and is structured with the aims of generating well-documented databases, focused assessment of changes over an extended period of time using standardized exposure conditions noting that most tests are conducted over periods of less than 1–2 years and thus do not enable true assessment of mechanisms activated due to longer-term exposures, and the development of a more comprehensive understanding of mechanisms of long-term durability so as to enable a better determination of design thresholds. This study is thus only part of a larger, and longer-term, focus.

## 2. Materials and Test Methods

Three different commercially available prefabricated CFRP composite strips used for the external strengthening of concrete structures through external bonding were investigated in this study. Two of the three systems are fabricated using the pultrusion process, whereas the third (system C in Table 1) is processed using a pulforming method. Details related to the three strips are given in Table 1.

Fiber volume fractions were determined through the use of acid-digestion, and glass transition temperatures were determined through dynamic mechanical thermal analysis (DMTA) in three-point bend mode using the peak of the tan delta curve as the measure of determination. The test was conducted at a frequency of 1 hz at a heating rate of 5 °C/minute between 25 °C and 250 °C. For the purposes of the current investigation the prefabricated strips were characterized in tension following ASTM D3039 [43] and Short-Beam-Shear (SBS) following ASTM D2344 [44], using 5 specimens in each case to ensure sufficient data to assess scatter bounds. Tensile tests were conducted on specimens of 254 mm length and 12.7 mm width with a gauge length of 200 mm using emery cloth at ends to ensure gripping and uniform distribution of stress from load grips. Tests were conducted at a constant crosshead speed of 2 mm/min with testing continued till failure. SBS tests were conducted on specimens with a span to thickness ratio of 5:1 to allow for the lower specimen thicknesses with a width of 4 mm and a loading rate of 1 mm/min. All specimens were inspected after cutting and those with longitudinal or edge cracks or other surface flaws/inconsistencies were rejected. While the primary characteristics of interest in rehabilitation of concrete structures are the tensile strength and modulus (or strain-to-failure) of the composite the inclusion of SBS strength is to enable assessment of degradation within the composite at the intralaminar level primarily due to fiber–matrix debonding and matrix deterioration due to long-term aging with associated ingress of solutions. This is especially important since the composites under consideration are unidirectional in nature and hence are more likely to indicate interface and matrix level changes (both of which could significantly affect overall effectiveness in the long-term) through an interfacial/intralaminar test, such as the SBS test, than the traditional longitudinal tensile test.

Composite response over time is affected by a range of factors including temperature, immersion in solution, relative humidity, UV, sustained load levels, cyclic/fatigue loading, and combinations thereof. This investigation considers a subset of these acknowledging that the selected exposures are not comprehensive but that they would provide the fundamental basis for future more complex analysis. Once cut to the required dimensions, all specimens were preconditioned for 30 days at 23 °C and 30% relative humidity to ensure a uniform baseline. Specimens were then exposed to the following 6 environments for periods of up to 5 years.

(a) Ambient exposure consisting of controlled conditions of 23 °C
(b) Immersion in deionized water at 23 °C (73 °F)
(c) Immersion in deionized water at 37.8 °C (100 °F)
(d) Immersion in deionized water at 60 °C (140 °F)
(e) Immersion in saltwater (5% NaCl solution) at 23 °C
(f) Immersion in concrete based alkaline solution at 23 °C. This solution was developed by immersing concrete discs of 25.4 mm height and 150 mm diameter in water to achieve the required pH and ionic content representative of a solution likely to be seen at the interface between the concrete substrate and the externally bonded FRP as detailed by Karbhari et al. [45]. The pH was 10.2 with ionic concentration as reported earlier in [45].

All specimens subject to immersion were placed in water baths such that there was no contact between adjacent specimens thereby ensuring full exposure to solution. The baths were maintained at the required temperatures maintained to within ±3 °C. A minimum of 5 specimens were tested at each condition at periods of 2, 6, 12, 18, 24, 36, 48 and 60 months. The ambient exposure specimens were tested at the same time as others to enable assessment of changes over time, if any, and to build a larger set of results that could be used for statistical analysis of the unexposed composites. Immersion in water at 23 °C was selected as being representative of a harsh, but realistic, exposure environment. It is acknowledged that results accruing from this exposure are likely to indicate a higher level of deterioration than exposure to humidity but could be considered a conservative threshold. The use of the two elevated temperatures of immersion (37.8 °C and 60 °C) allowed for accelerated aging, whereas the other two solutions provided the range of environments that could be faced by rehabilitation of marine/offshore structures as well as the effects of moisture transport to the composite through the concrete element which could hence be expected to have a ionic makeup and concentration similar to that of pore water in concrete. The levels of elevated temperature used in this investigation, as related to the condition of immersion in deionized water, are significantly below the glass transition temperatures of the three systems and are hence not expected to result in degradation associated with mechanisms initiated/accelerated by proximity to the T_g_. Thus, effects will not be induced by T_g_ related softening/deterioration of the matrix.

## 3. Results and Discussion

### 3.1. Unexposed Specimens

Results of baseline characteristics are shown in Table 2. It should be noted that the averages and standard deviations are in line with those reported previously for similar systems by Wang et al. [46] with an average tensile strength and modulus of 3133.4 MPa and 191.6 GPa, with standard deviations of 87.3 MPa and 2.62 GPa, respectively. Nguyen et al. [47] also reported average tensile strengths and moduli of 2389.4 MPa and 156.99 GPa, with standard deviations of 298.98 MPa and 9.82 GPa, respectively. Sika, in their datasheets for the CarboDur S System report mean and design tensile strengths of 3100 MPa and 2800 MPa, respectively, which indicates a standard deviation of 100 MPa, and a mean and design tensile modulus of 165 GPa and 160 GPa, indicating a standard deviation of 1.67 GPa [48].

As can be seen, system A has the highest tensile strength and modulus while system C has the lowest. While it may, at first glance, appear that the difference can be attributed completely to fiber volume fraction a simple constituent analysis using the rule-of-mixtures indicates that there are further reasons including differences in fiber–matrix interface bonding and fiber linearity, which can also be noted in SBS results. This can be directly attributed to the manufacturing method itself since the tension in pultrusion causes fibers to remain aligned whereas the use of a two-step process of preforming followed by processing in pulforming results in non-linearities that result in a drop in both strength and modulus. A comparison shows that system C has levels of tensile strength, modulus, and SBS strength 12.3%, 25.8% and 8.11% less than system A with the significantly lower modulus being an indicator of non-linearity effects. In contrast, the values of system B, which has a volume fraction of 61% compared to the 69% level of system A, are more in line with the effects of volume fraction. Further, the decrease in SBS strength of system A as compared to system B is in line with the effect of high fiber volume fraction on interfacial response as detailed by Karbhari and Xian [49].

Tests on specimens maintained at 23 °C through the 5-year (60-month) period of investigation showed very little change in all characteristics indicating that, as expected, the specimens were completely cured at the outset and did not undergo posture, or deterioration, over time. For purposes of design the average values as tested are rarely used. Rather, the design value is chosen to be conservative and represents the minimum performance level a material can be expected to maintain, providing a lower bound for its performance. Within the aerospace sector, design values for composites are determined using either the A-basis (defined as the lower 1st percentile of a population distribution with 95% confidence) or the B-basis (defined as the lower 10th percentile of the population with a 95% confidence [50]. In the case of externally bonded FRP strips for the rehabilitation of concrete ACI-440 [17,30] defines the guaranteed performance level as

**Figure 1:**
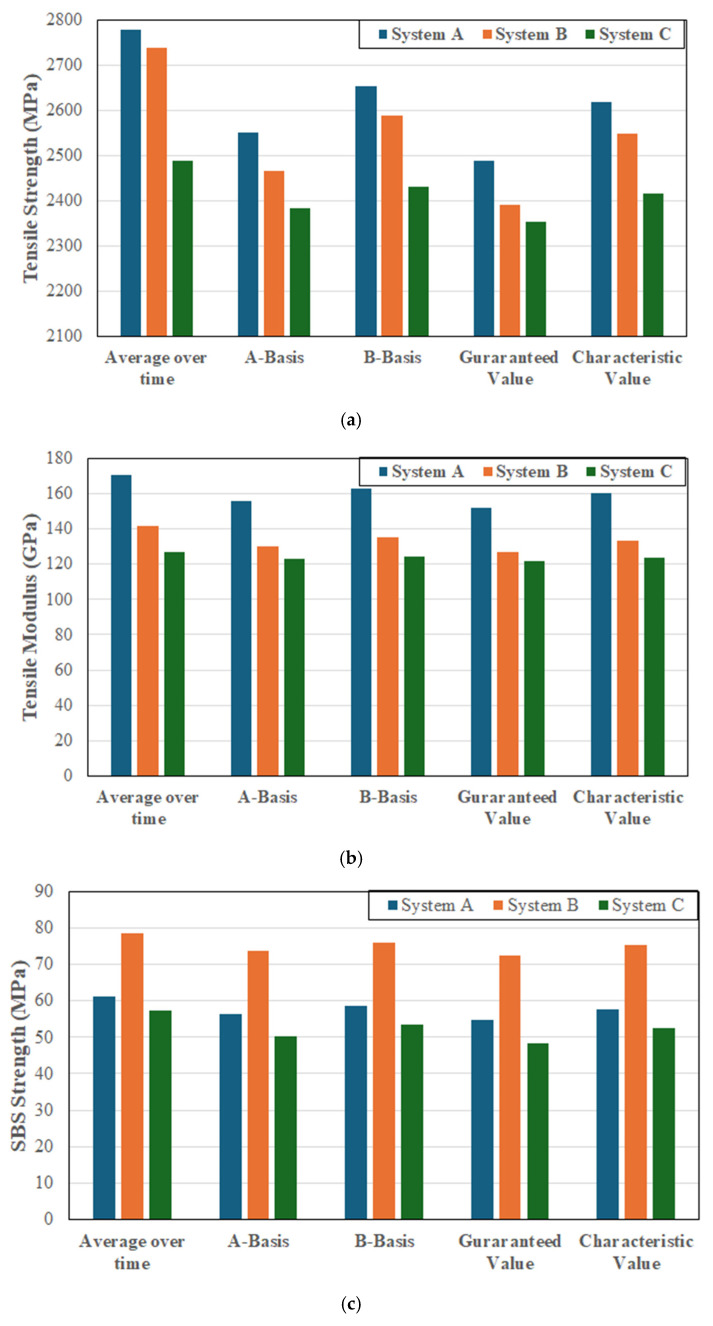
(**a**): Performance attributes and thresholds related to tensile strength. The mean and standard deviation for systems A, B, and C, are 2778.48 MPa and 96.97 MPa, 2736.98 MPa and 115.02 MPa, and 2489.53 MPa and 45.22 MPa, respectively. (**b**): Performance attributes and thresholds related to tensile modulus. The mean and standard deviation for systems A, B, and C, are 170.58 GPa and 6.22 GPa, 141.47 GPa and 4.86 GPa, and 126.62 GPa and 1.65 GPa, respectively. (**c**): Performance attributes and thresholds related to short beam shear strength. The mean and standard deviation for systems A, B, and C, are 61.31 MPa and 2.15 MPa, 78.37 MPa and 1.98 MPa, and 57.4 MPa and 2.98 MPa, respectively.

(1) Guaranteed Level=μ−nσ

where m is the sample mean of the tested property, *n* (=3) is the multiplier corresponding to the required statistical confidence level, and s is the sample standard deviation. The value of 3 used for the multiplier in Equation (1) corresponds to a statistical confidence level of approximately 99.87% under the assumption of a normal distribution. This value is slightly more conservative than the B-basis which uses a multiplier of 1.66 in comparison. Both the Canadian Highway Bridge Design Code [51] and the fiB model code [52] define the characteristic value as the 5th percentile of the population with a 95% confidence level, such that *n* = 1.645. Overall results for the three systems as related to population mean, A-basis, B-basis, guaranteed performance level, and the characteristic value for the tensile strength and modulus and the SBS strength are given in Figure 1a–c. The A- and B-basis values are used extensively in the aerospace and defense sectors with composites and are hence provided here as a basis of comparison since they are predominantly used with extremely well characterized and highly regulated materials systems which have to be extensively qualified, including at the constituent (fiber and matrix) and process levels [50].

As expected the guaranteed values are the lowest of all the sets determined for each of the three FRP systems, with the guaranteed values for system A being 89.5%, 89.1% and 89.5% of the mean for tensile strength, tensile modulus and SBS strength, respectively, while those for system B are 87.4%, 89.7% and 92.4%, respectively, and the corresponding values for system C are 94.6%, 96.1%, and 84.4%, respectively, suggesting that on a preliminary basis systems A and B are similar, but both are different from system C.

Given that the design values are often based on the assumption of normality of the distribution of populations, and the Weibull distribution has been widely used in describing failure of composites [53,54], it is of interest to assess the fit of these data to these two distributions. The probability density function (PDF) for the normal distribution is expressed as

(2) $$f\left(x\right)=\frac{1}{\sigma \sqrt{2\pi }}\;e^{-\frac{1}{2}{\left(\frac{x-\mu }{\sigma }\right)}^{2}}$$

where m is the mean, and s is the standard deviation. The 2-parameter Weibull distribution is expressed as

(3) $$f\left(x\right)=\frac{\alpha }{\beta }{\left(\frac{x}{\beta }\right)}^{\alpha -1}e^{-{\left(\frac{x}{\beta }\right)}^{\alpha }}$$

where a and b are the shape and scale parameters, respectively. Values of these parameters are given in Table 2 for the normal distribution and Table 3 for the Weibull distribution.

In the normal distribution the standard deviation, s, is a direct measure of scatter with a larger standard deviation being indicative of greater scatter around the mean. In comparison the shape parameter, a, governs the form and concentration of the distribution with a high value of a representing a narrower peak and hence less scatter, and a lower value of a being indicative of a flatter or more spread-out distribution representative of greater scatter. The scale parameter, b, describes the relative dispersion and sets the location with a higher value increasing the absolute value but not necessarily the relative scatter. As can be seen from a comparison of values in Table 2 and Table 3 the scale parameter in Table 3 is analogous to the average and in all cases is higher than the mean for the normal distribution in Table 2. The shape parameter, however, does not correlate directly with the standard deviation. For example, scatter in tensile modulus as indicated by the standard deviation is the highest for set B and the lowest for set C, whereas set C has the highest value of a and hence the least scatter. It is of interest to note that for tensile strength scatter as denoted by the shape parameter, a, follows the trend of C < A < B, whereas the order for tensile modulus is C < B < A and that for SBS strength is B < A < C.

In addition to the Kolmogorov–Smirnov test which was used to assess both normal and Weibull fits, the Shapiro–Wilk test was used to assess normality. Results are summarized in Table 4.

It should be noted that the Shapiro–Wilk test uses a value of 0.05 to assess normality, i.e., if *p* > 0.05 the null hypothesis is not rejected and the dataset is consistent with a normal distribution. In this case population sets with *p* = 0.011 and *p* = 0.003, representative of FRP systems A and B for tensile strength, indicate significant deviation from normality. This is confirmed by the Kolmogorov–Smirnov test where the *p* value for the normal distribution is significantly lower than that for the Weibull distribution indicating that the latter provides the better fit. Overall, it is clear that neither distribution can be used across all three systems emphasizing the lack of qualification through rigorous specifications as would be necessitated in the aerospace/defense sectors. In the case of FRP system C for SBS strength, while the Shapiro–Wilk test indicates normality, the Kolmogorov–Smirnov test statistic is lower for the Weibull distribution and the *p*-value is higher for the Weibull distribution suggesting that while both distributions could be used the Weibull provides the better fit. Knowledge of the fits and parameters are useful for further analysis using reliability-based approaches [55,56]. A comparison of distribution fits across the three systems indicates that systems A and B show similarities in the preferred distributions whereas system C is again different, following the conclusion reached earlier on comparison of basis and guaranteed values.

While FRP products are currently available without a preset standard or specification for the product, resulting in a variation in both geometrical (such as thickness in the case of the three prefabricated FRP strips considered in the current investigation) and performance characteristics, it would be advantageous for the systems to meet specific thresholds that could then be used generically for design in a manner similar to that for structural steel. An assessment by pooling data for all three systems and use of the Shapiro–Wilk test indicates significant non-normality for all three characteristics (tensile strength, tensile modulus, and SBS strength) with the Kolmogorov–Smirnov tests indicating a slight preference for the Weibull distribution on tensile strength but poor fits for both normal and Weibull distributions for tensile modulus and SBS strength with indications of multimodal behavior. This is not unexpected given the differences among the three systems as noted earlier. It is, however, of interest to note that pooling of only Sets A and B results in a similar conclusion with the tensile strength fitting the Weibull distribution extremely well (*p* = 0.919 compared to 0.0079 for all three sets together) but neither showing a good fit for the tensile modulus and SBS strength. Thus, significant work remains for the development of a performance-based standard for these products for use in general applications as has been achieved for structural steel in civil infrastructure applications and prepreg based autoclave cured composites in aerospace and defense applications.

### 3.2. Effect of Hydrothermal Exposure

**Figure 2:**
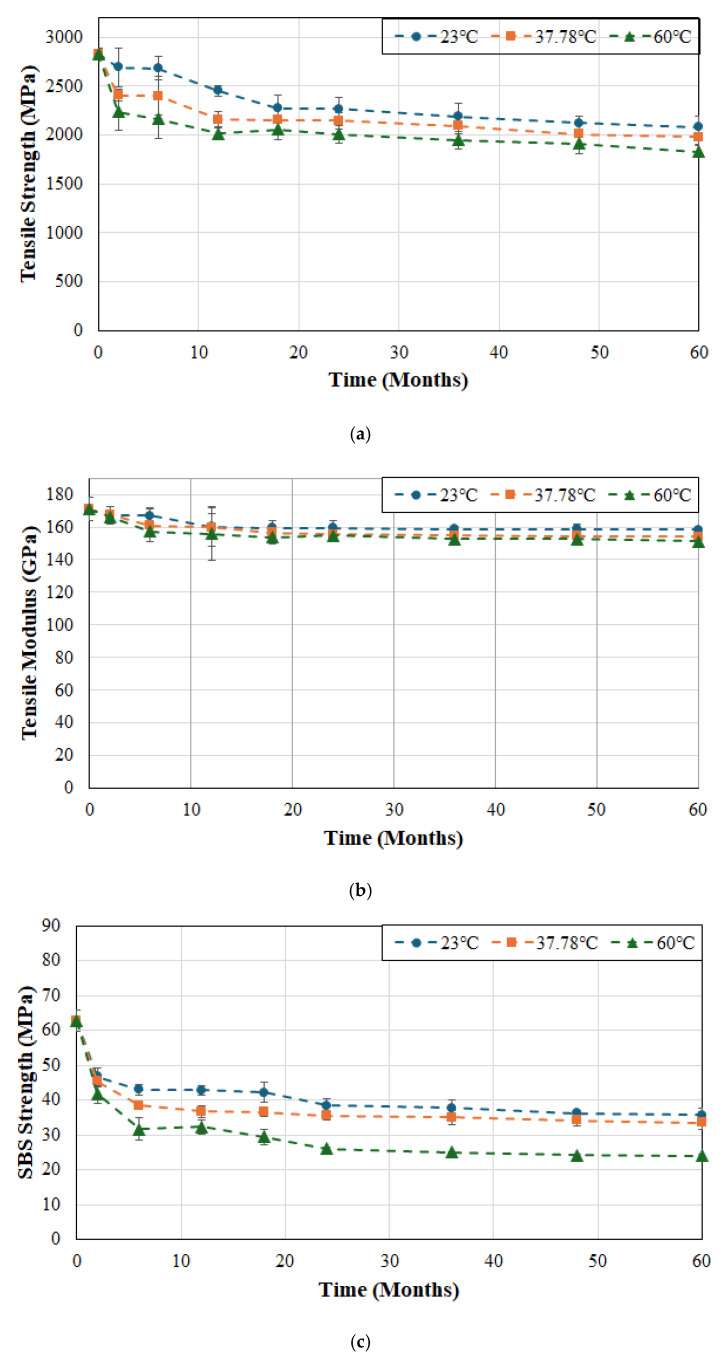
(**a**): Change in tensile strength of FRP system A as a function of temperature and time of immersion in deionized water. (**b**): Change in tensile modulus of FRP system A as a function of temperature and time of immersion in deionized water. (**c**): Change in SBS strength of FRP system A as a function of temperature and time of immersion in deionized water.

Since the resin and fiber–matrix interface are susceptible to degradation as a result of moisture uptake, tests were conducted by immersing specimens in deionized water as representative of exposure in a moist/humid environment. In order to assess the synergistic effects of heat and solution, tests were conducted using three different temperatures of 23, 37.8 and 60 °C, which not only enabled assessment of hydrothermal effects but also for the use of time–temperature superposition principles for prediction of long-term durability of these materials. It is expected that increasing the temperature of immersion would accelerate deterioration of the matrix, and the fiber–matrix interface. Effects of hydrothermal ageing on tensile strength, tensile modulus, and SBS strength for FRP system A are shown in Figure 2a, Figure 2b and Figure 2c, respectively.

As can be seen from Figure 2a the largest drop in tensile strength occurs in the first two months of immersion with rates being 2.39%, 7.47%, and 10.47% per month at 23, 37.8 and 60 °C, respectively, suggesting rapid fluid ingress and attack of the interface. The initial drop is followed by a short period (from 2 to 6 months) of significantly lower rates of deterioration (0.1%, 0.1% and 0.81% per month at the three temperatures) suggestive of initial attainment of moisture saturation. This is followed by an increase which, in the case of water at 23 °C, continues linearly from 6 to 18 months whereas at the two higher temperatures it occurs for a shorter period of 6–12 months, after which all three cases show slow continuing degradation with the rate of deterioration decreasing with increase in time except at the highest temperature of immersion. It is of interest to note that the deterioration at the end of the first 12-month period is 13.4%, 23.6% and 27.7% for the cases of immersion in deionized water at 23, 37.8 and 60 °C, respectively, which is a significant part of the overall deterioration in tensile strength at the end of 60 months of 26.4%, 30% and 35.3%, at the three temperatures of immersion. It is clear that an increase in temperature not only increases the level of deterioration, but also the overall rate. Similar results have been reported earlier for wet layup based carbon/epoxy strengthening systems [57].

While the retention level of tensile strength depends to an extent on the integrity of the interface and the ability to transfer stresses from fibers to the matrix, the modulus of a unidirectional composite is fiber dominated and thus will result in a significantly lower level of degradation through environmental exposure, as is seen in Figure 2b. It should be noted that moisture sorption primarily affects the resin and the interface rather than carbon fibers and, hence, the longitudinal modulus of a unidirectional composite is affected significantly less than its longitudinal tensile strength or SBs strength, and decreases result primarily from the degradation of the resin and interface. Zafar et al. found that the tensile modulus of an epoxy decreased by about 7.5% after immersion in water for 300 days compared to a reduction of 15–18% in its strength [58]. There is a clear differentiation in the rate of degradation over the initial period of 2–6 months where rates are about 1%/month at 23 and 37.8 °C and 1.3–1.4%/month at 60 °C, after which rates decrease substantially. The level of degradation over the first 12 months is 6.1%, 6.5% and 8.6% for the cases of immersion in deionized water at 23, 37.8 and 60 °C, respectively, with the final level of degradation at the end of the 60-month period of investigation being 7.3%, 9.8% and 11.4%, at the three temperature levels. It is emphasized that the level of decrease in tensile modulus is significantly less than that that in tensile strength, which is in line with previously reported studies [57,59].

As seen in Figure 2c the SBS strength results are similar to those of the tensile strength in that there is a rapid decline in the first two months with the major drop in the first six months after which there is a slow increase in the level of deterioration. This can be linked to the predominance of the interfacial bonding mechanisms in driving deterioration as reported earlier [57,60]. Although the tensile and SBS strengths decline at a faster rate at higher temperatures of immersion, studies have shown that after the initial period the curves present asymptotic trends [36] with comparable overall profiles because of the initial water induced interfacial degradation [59]. There is similarity in the modes of degradation, especially when the fiber volume fraction is high, which directly restricts the volume of bulk matrix and hence mechanisms therein. However, the extent of deterioration is significantly greater, emphasizing the effect of loss of interfacial bond integrity. It is of interest to note that the effect of the initial increase in temperature is minimal with the decrease at the end of the 60-month period of immersion at 37.8 °C is 46.6% versus 43.1% at 23 °C and a much greater level of 61.6% at 60 °C. The sharp initial drop in the first six months is related to diffusion and plasticization with the slower decline being due to longer-term hydrolysis and increased interface degradation and microcracking. Hong et al. [55] reported the significantly greater drops in interlaminar strength in comparison to the fiber-dominated tensile strength and emphasized the need to include the assessment of SBS strength as an important aspect in determination of long-term durability.

**Figure 3:**
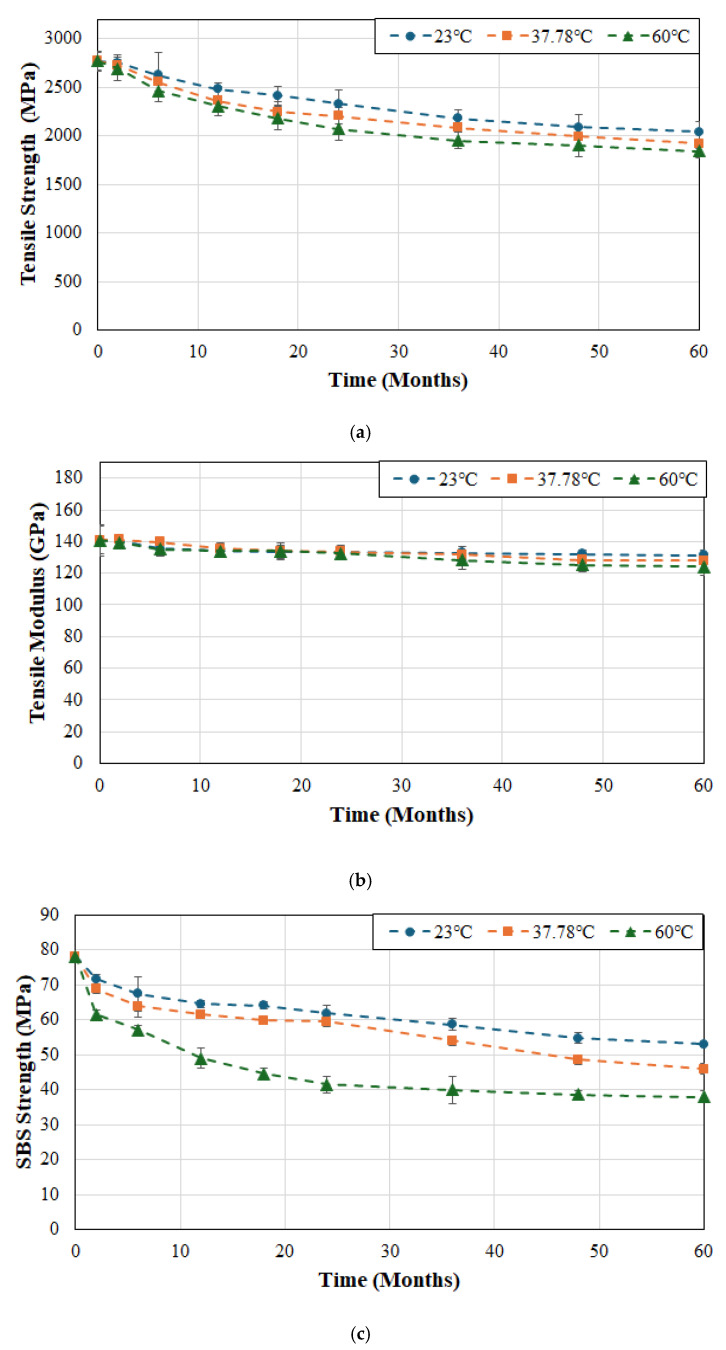
(**a**): Change in tensile strength of FRP system B as a function of temperature and time of immersion in deionized water. (**b**): Change in tensile modulus of FRP system B as a function of temperature and time of immersion in deionized water. (**c**): Change in SBS strength of FRP system B as a function of temperature and time of immersion in deionized water.

The effects of temperature of immersion on tensile strength, tensile modulus, and SBS strength of system B are shown in Figure 3a, Figure 3b and Figure 3c, respectively. In comparison to system A the decrease in strength appears less rapid and extends over a longer period of time without the sudden initial drop suggesting slower uptake of moisture and hence decreased initial plasticization which is confirmed by the earlier studies by Karbhari and Ghosh [61] on the same material systems. It is emphasized that the initial uptake of water, accelerated by an increase in temperature, leads to the weakening of inter-chain van der Waals forces and hydrogen bonds leading to plasticization and, hence, a decrease in mechanical performance [62]. The overall drop in tensile strength after 6 months of exposure was 26.4%, 30.6%, and 33.4% for the three temperature levels of 23, 37.8 and 60 °C, which is very close to that of system A but follows a more gradual progression with time. This can be traced to the higher matrix volume fraction in system B which results in slower interfacial failure, which is in line with earlier results [63,64] showing that SBS strength decreases consistently with increase in fiber volume fraction. Overall, the drop in tensile modulus is fairly low at 6.7%, 9% and 11.9% at temperature levels of 23, 37.8 and 60 °C, respectively, roughly similar to that seen in system A. Given the more gradual drop in tensile strength it is expected that the SBS strength would also show a reduced level of deterioration. It is interesting to note that at the two lower temperatures the drop is continuous and gradual but at the highest temperature, 60 °C, the multi-phase response consists of an initial stage that shows rapid decrease attributed to plasticization and interface based capillarity, which is followed by a slower decrease, and often a third stage extending to an asymptotic trend over longer periods of exposure. In the current case there is a rapid drop of 21.3% in the first two months followed by an overall drop of 51.3% over the 60-month period of immersion. In comparison, at the two lower temperatures, 23 and 37.8 °C, the drop in the first two months was 8.3% and 12%, respectively, and that at the end of the 60-month period was 32.1% and 41.2%, respectively, with all levels showing levels of decline less than those of system A. The distinct difference in drops of tensile and SBS strength from the beginning of exposure with temperature of immersion follows similar trends reported by Liu et al. [36] for SBS strength where they noted that the rates corresponded to the accelerated rates of diffusion with temperature, and that as saturation was attained the deteriorative effects on the interface weaken resulting in asymptotic response.

**Figure 4:**
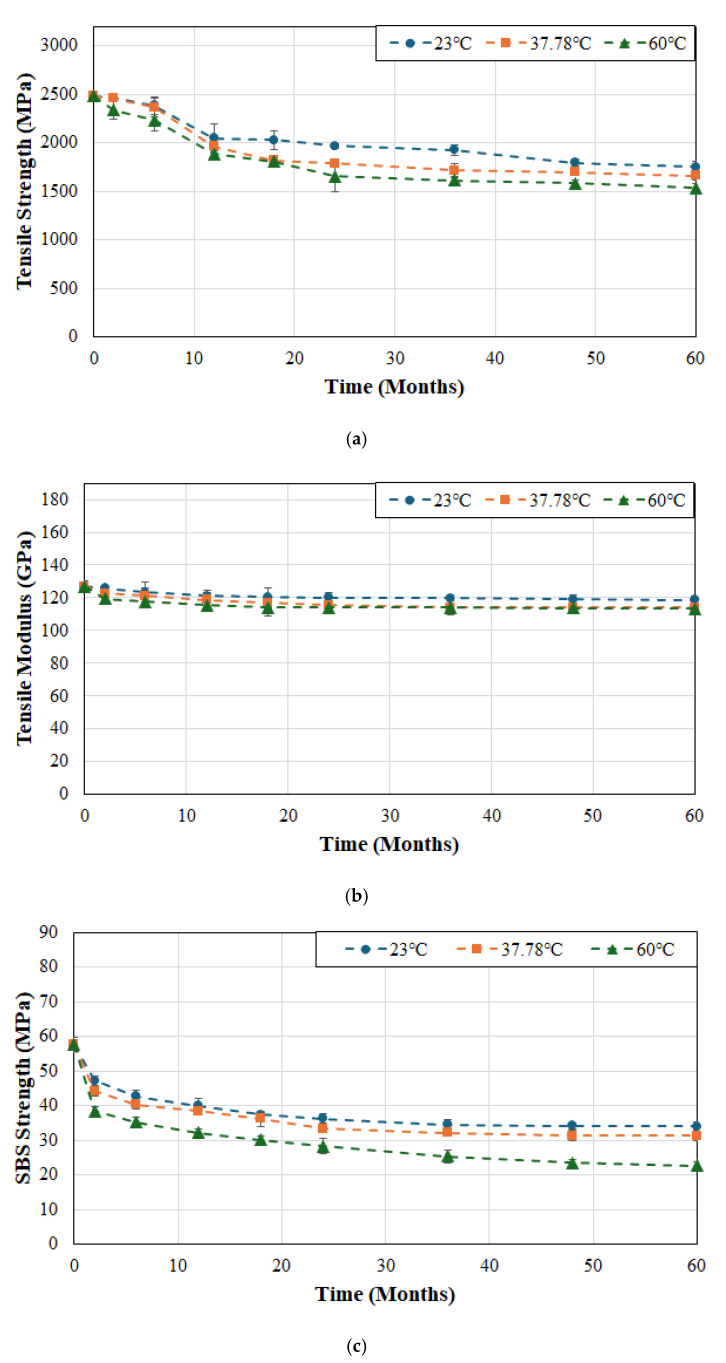
(**a**): Change in tensile strength of FRP system C as a function of temperature and time of immersion in deionized water. (**b**): Change in tensile modulus of FRP system C as a function of temperature and time of immersion in deionized water. (**c**): Change in SBS strength of FRP system C as a function of temperature and time of immersion in deionized water.

As seen in Figure 4a the tensile strength for system C does not degrade significantly over the first 6 weeks of immersion at the two lower temperatures of 23 and 37.8 °C with drops of only 4% and 4.6% as compared to 10% after immersion in deionized water at 60 °C. The total loss at the end of the 60-month period of immersion was 29.4%, 33.2% and 38.2% at temperatures of 23, 37.8 and 60 °C, respectively. It is of interest to note that the rate of loss past the 24 month period of immersion is extremely low and fairly stable till the end of the overall period of immersion with drops of 10.9%, 7.2% and 7.4% over this time period for cases of immersion at 23, 37.8 and 60 °C, respectively. While it might appear counterintuitive for the drop to be greater at the lowest temperature of immersion it should be remembered that the deteriorative mechanisms are initially accelerated at the higher temperatures of immersion and thus the reactions at 23 °C are slower and the effects accrue over a longer period of time. As with the two other systems, the drop in modulus is significantly less at 6.3%, 10.2% and 10.7% for cases of immersion at 23, 37.8 and 60 °C, respectively, with the level of decrease being extremely slow and asymptotic after 12 months of immersion at which point the reductions were 4.3% (i.e., 68.3% of the overall decrease), 6.5% (i.e., 63.7% of the overall decrease) and 9% (i.e., 84.1% of the overall decrease) emphasizing that the maximum decrease occurs in the initial phase of moisture uptake due to plasticization and is largely reversible as reported earlier [49]. The SBS strength shows a rapid decrease in the first 2 months (with drops of 18.2%, 23.4%, and 33.4% due to immersion in deionized water at 23, 37.8 and 60 °C, respectively), followed by a more gradual decrease till the end of the 60-month period, representative of damage saturation and slower hydrolysis and interface shear weakening. The initial plasticization is accompanied by faster kinetics of matrix and interface degradation at the higher temperatures of immersion with the deterioration continuing at a faster rate till the end due to the acceleration of thermally activated chemical and interface breakdown. It should be noted that over the last 12 months of immersion the rate of deterioration in SBS strength was 0.19%/month at the two lower temperatures but 0.26%/month at 60 °C, a 36.8% increase. The continued change in mechanical properties even after the point where saturation of moisture uptake can be expected follows the results reported by Ghabezi and Harrison [65] who hypothesized that the effects of chemical based degradation can be higher than those due to moisture induced stresses and mechanisms.

**Figure 5:**
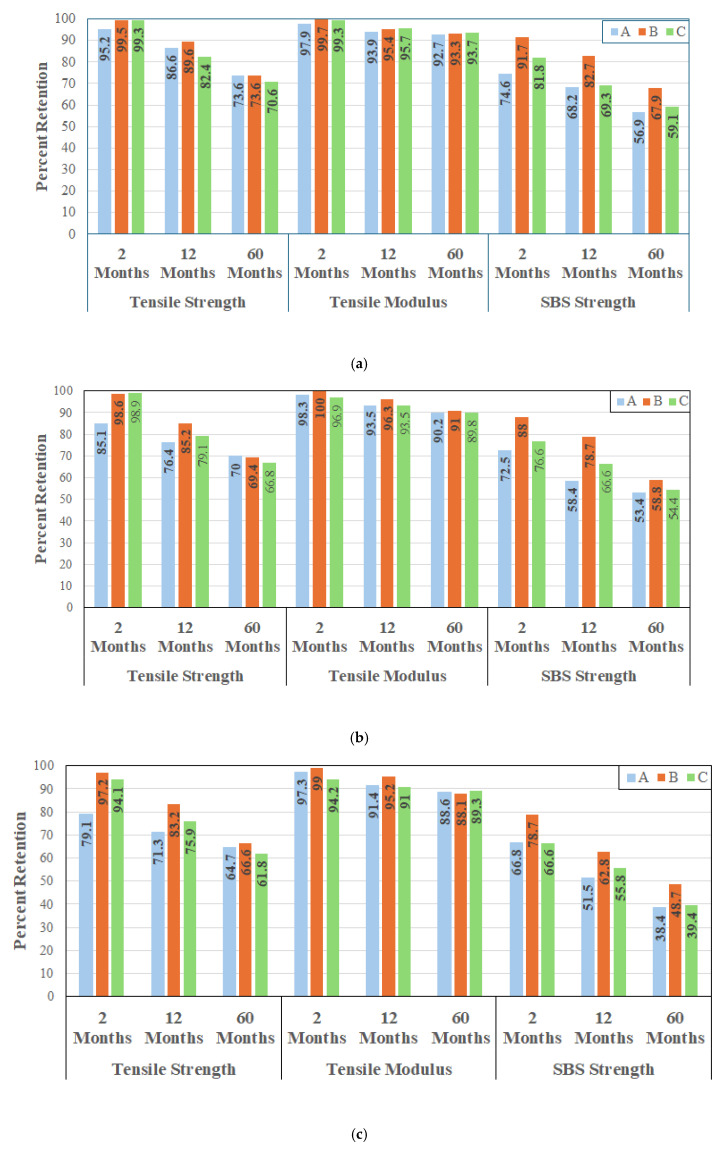
(**a**): Retention of tensile strength, tensile modulus and SBS strength after immersion in deionized water at 23 °C. (**b**): Retention of tensile strength, tensile modulus and SBS strength after immersion in deionized water at 37.8 °C. (**c**): Retention of tensile strength, tensile modulus and SBS strength after immersion in deionized water at 60 °C.

It is clear that across all three systems an increase in temperature of immersion results in an acceleration of deterioration in performance with the greatest level of decrease being seen in SBS strength and the least in tensile modulus, which is in line with the characteristics of unidirectional carbon fiber reinforced composites processed at moderately high temperatures with fiber volume fractions above 60%. A comparison of performance retention of tensile strength, tensile modulus, and SBS strengths for the three systems as a result of immersion in deionized water at 23, 37.8 and 60 °C is shown in Figure 5a, Figure 5b and Figure 5c, respectively. Given that the overall response can generically be divided into three zones with the initial being for the first two months, where effects are primarily due to initial moisture uptake and plasticization, the period from 2–12 months where there is hydrolysis and interface degradation, and then for the longer term up to 60 months where change is relatively slower and largely asymptotic, results are compared at the 2-month, 12-month, and 60-month levels for ease of comparison.

As can be seen from Figure 5a, system A shows the least retention after 2 months of immersion in deionized water at 23 °C across all three performance characteristics. systems B and C show extremely similar retention levels for tensile strength and modulus at this time period but system C shows greater deterioration in SBS strength. At the 12-month level system C shows the least retention for tensile strength and is similar to that of system A for both tensile modulus and SBS strength, both of which are lower than system B. At the end of the 60-month period of immersion the retention of tensile strength (at 70.6–73.6%) and modulus (92.7–93.7%) are similar across all three systems and well within scatter. However, the SBS strength retention is substantially higher for system B which has the lowest fiber volume fraction and hence greater amount of bulk matrix resulting in better wetout of fibers. It is noted that higher levels of degradation of SBS strength are attributed to greater fiber–matrix debonding [66] which would be decreased by better wetout.

Given this response, it is interesting to note that systems B and C both show very minor changes in levels of retention of tensile strength as the temperature of immersion is increased from 23 °C to 37.8 °C, while system A, which had a much higher fiber volume fraction, shows a significant drop in retention from 95.2% to 85.1% at these temperatures. This can be attributed to the greater propensity for capillary action of moisture transport between fibers that were perhaps not as well wet out in the higher fiber volume fraction material set [49] and due to the greater number of voids that could exist at higher fiber volume fraction levels [67] due to dry spots within fiber tow/bundles with insufficient wetout. Interestingly, at the 12-month level the increased temperature results in a drop of 3.6%, 4.2%, and 3.8% from levels seen at 23 °C for systems A, B, and C, respectively, with system C showing the lowest retention. The trends are similar for retention of tensile modulus with the lowest retention again being from system C at 89.8% which is a 3.9% drop from that noted at 23 °C. Systems A and B in comparison show lesser drops of 2.5% and 2.3%, respectively. While system B still shows the highest retention in SBS strength, the differences between the three systems after 60 months of immersion at this temperature are much smaller emphasizing the effects of plasticization in the resin after initial fiber–matrix debonding. While systems A and C showed a further drop of 3.5% and 4.7%, respectively, from that due to immersion in deionized water at 23 °C, system B showed a much larger drop of 9.4% from a retention level of 67.9% to 58.5%. At the highest temperature of immersion (60 °C) the deterioration is further accelerated with significant drops in both tensile strength and SBS strength. However, the tensile modulus, which is a fiber dominated characteristic, remains largely unaffected with the 60-month retention levels being 88.6%, 88.1% and 89.3% in comparison to levels of 92.7%, 93.3% and 93.7% at 23 °C. As with the other two temperatures, the three systems show very similar levels of retention at 60 °C, which can be traced to the dominant effects of carbon fibers in the longitudinal direction which are unaffected by deionized water. It is of interest to note that between 12 and 60 months, the averaged rates of decrease in tensile strength were 0.19%/month, 0.42%/month and 0.39%/month for systems A, B and C, respectively, indicating that while system A may have shown a larger decrease in the first phase (2 months) the overall system had better retention due to higher fiber volume fraction even after accelerated capillary action as related to moisture uptake. It is noted that an earlier investigation on the same systems reported significantly higher moisture uptake levels for system A in comparison to systems B and C which had about half the uptake [61]. These results should hence be considered with caution in that even though there may be good retention in tensile strength and modulus under accelerated conditions of aging, the overall deterioration of the composite, especially at the interfacial and intralaminar level could be significantly higher as noted previously [60]. Thus, consideration of only fiber-dominated characteristics of performance could result in misleading [59] and non-conservative, predictions, and selection of design values.

**Figure 6:**
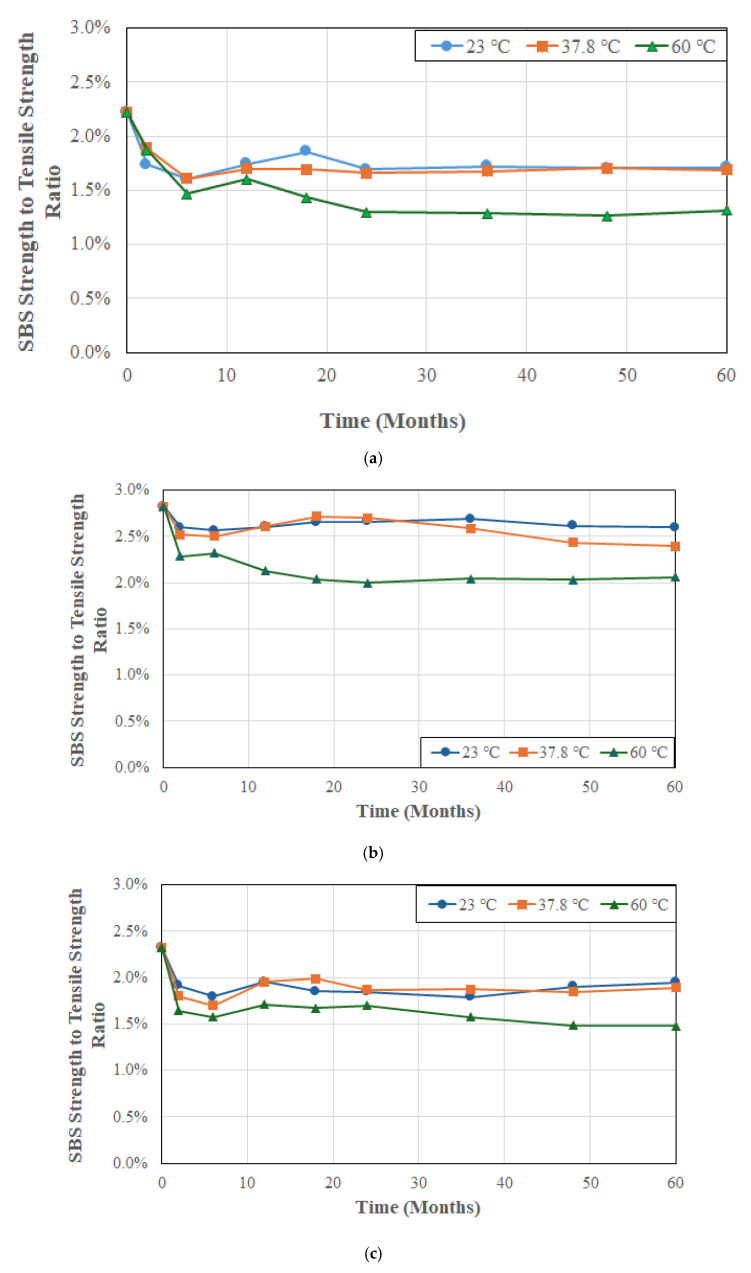
(**a**): Change in SBS strength to tensile strength ratio of system A as a function of temperature of immersion. (**b**): Change in SBS strength to tensile strength ratio of system B as a function of temperature of immersion. (**c**): Change in SBS strength to tensile strength ratio of system C as a function of temperature of immersion.

With this in mind, it is of interest to consider the ratio of SBS strength to tensile strength and its evolution with time, and temperature, of immersion in solution. The SBS strength is indicative of a materials resistance to interlaminar shear failure and is governed by the matrix-dominated behavior and the level of integrity of the fiber–matrix interface. On the other hand, the longitudinal tensile strength of a unidirectional composite reflects the effectiveness of stress transfer and hence the load-carrying capacity along the fiber direction, which is largely dominated by fiber strength characteristics. Thus, a low ratio indicates that the shear strength is much lower than the tensile strength, which is typical for carbon fiber reinforced unidirectional composites where matrix/interface failure governs the SBS strength. A higher ratio serves as an indication of better matrix or interfacial bonding, or composites designed with toughened matrices. The low ratio underscores a key design limitation of unidirectional composites in that they have excellent tensile characteristics in the direction of the fibers, but their through-thickness and shear performance are limited. This ratio is critical when designing against shear-driven failures especially as a function of exposure condition and time, with decreasing ratios indicative of greater level of degradation. It is thus of significant interest to assess the change in this ratio as a function of temperature of deionized water as shown in Figure 6a, Figure 6b, and Figure 6c for systems A, B, and C, respectively.

As can be seen from Figure 6a–c, the initial ratio is highest from system B at 2.82% compared to 2.22% for system A and 2.32% for system B. Cromwell et al. [68] conducted tensile and SBS tests on unidirectional carbon/epoxy plates with a ratio of 2.56% which falls within the range shown by the three systems being investigated in this study. All three systems show very little difference in response at the two lower temperatures of 23 and 37.8 °C, with the highest temperature of immersion resulting in a significant drop in ratio. This indicates that the overall effect of the two lower temperatures is similar but that the highest temperature results in significant matrix and interface level deterioration. System A shows a steep drop over the first 6 months whereas systems B and C have a smaller drop over the first 2 months. In all cases after these time periods the changes are slower.

As seen in Figure 6a the ratio for the three temperatures after the initial period of 6 months is similar with levels of 1.6% at both 23 and 37.8 °C and a lower level of 1.47% at the highest temperature of immersion of 60 °C. As a result of immersion at the highest temperature of 60 °C there is a drop from an initial level of 2.22% to 1.47% at 6 months followed by a slow decrease to 1.32% over the next 54 months, emphasizing the overall effect of initial plasticization and hydrolysis on overall hydrothermal damage. In the case of system B (Figure 6b) the ratios at the end of the 60-month period of immersion are 2.6%, 2.4%, and 2.1%, showing similarity of response due to the two lower temperatures as seen for system A and indicating greater matrix and interfacial degradation at the highest temperature. Figure 6c, which represents response of system C shows ratios of 1.95%, 1.89% and 1.48% at the three temperature levels of 23, 37.8, and 60 °C, respectively, showing similar trends.

**Figure 7:**
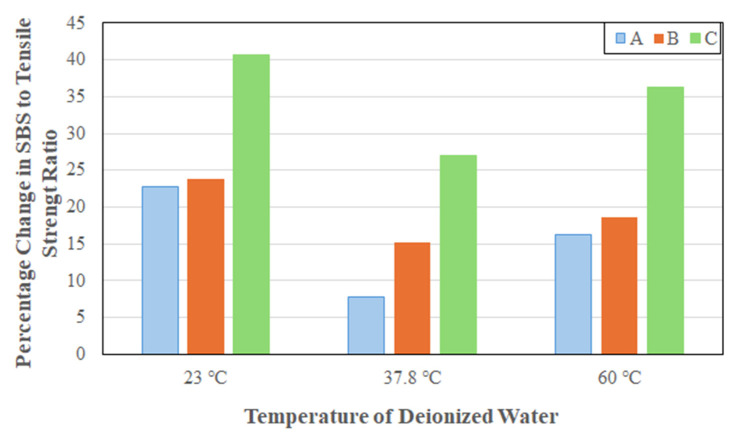
Change in SBS Strength to Tensile Strength Ratio Over 60 Months of Immersion as a Function of system and Temperature of Immersion.

As shown in Figure 7, overall system A shows the highest overall drop at all three temperatures with system B showing the lowest. The overall response of a decrease in ratio with increasing time and temperature of immersion indicates that the interlaminar shear capacity is degrading faster than the tensile strength in the longitudinal direction. The initial rapid drop suggests matrix softening due to plasticization which leads to lower interlaminar shear strength. This is followed by a short period of continued decrease albeit at a slightly lower rate due to fiber–matrix debonding associated with moisture uptake and matrix microcracking. Over the longer-term, mechanisms of hydrolysis and increased fiber–matrix debonding result in continued deterioration at a much slower rate. This type of behavior suggests a reduced durability margin and indicates changes in failure modes with early-life failures being more predictable due to fiber-dominated mechanisms, but prolonged exposure leading to resin or interface-dominated failures. This again has implications for both prediction of long-term durability and the development of safety factors for design whereby there may be need to consider interlaminar properties in addition to the more commonly used in-plane ones for characteristics such as tensile strength and modulus for assessment of environmental durability.

### 3.3. Effect of Solution

**Figure 8:**
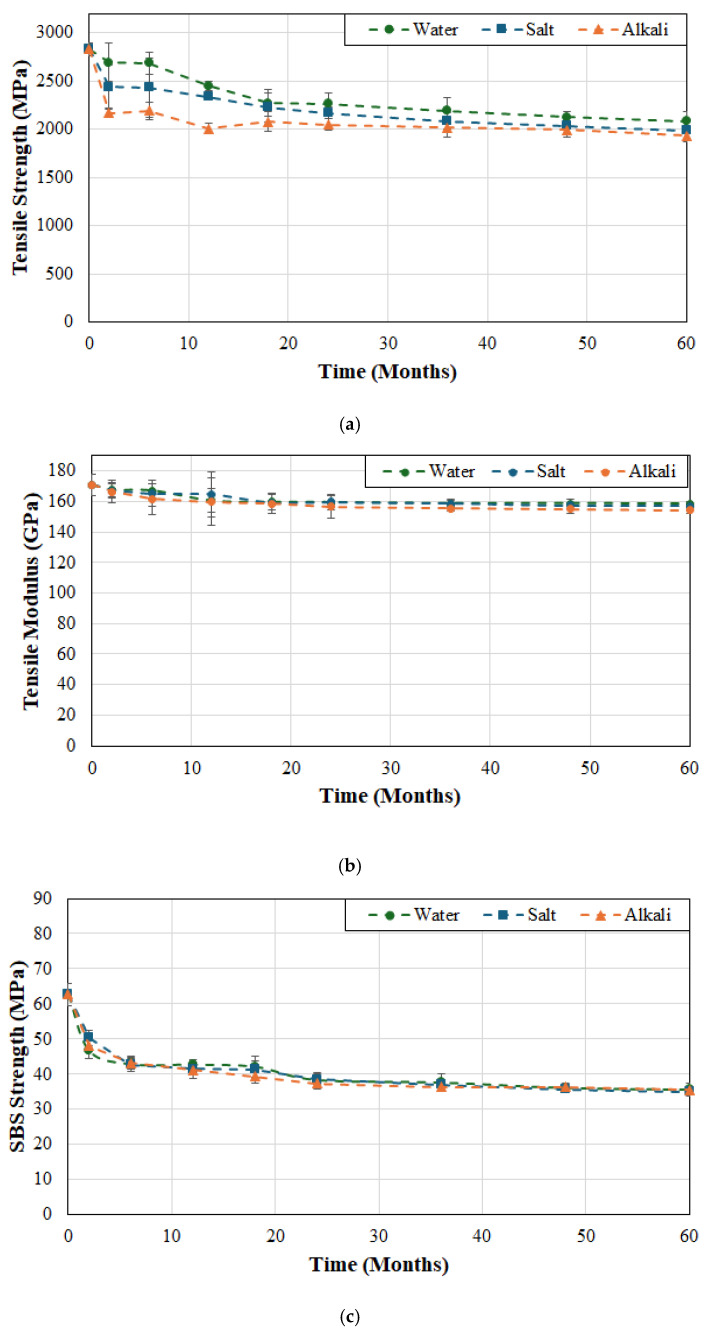
(**a**): Change in Tensile Strength of FRP system A as a Function of Solution Type, and Time, of Immersion. (**b**): Change in Tensile Modulus of FRP system A as a Function of Solution Type, and Time, of Immersion. (**c**): Change in SBS Strength of FRP system A as a Function of Solution Type, and Time, of Immersion.

Given the increasing use of external bonded FRP in rehabilitation/strengthening of marine infrastructure where the FRP strip is likely to be either immersed in sea water or be exposed to salt spray, and the cases where water could flow through the concrete structure to the FRP composite there is need to assess differences, if any, between the effects of water and those accruing from seawater and an alkali solution similar to that of pore water in concrete. Figure 8a–c shows the differences accruing from the type of solution on tensile strength, modulus and SBS strength over the period of 60 months.

As can be seen from Figure 8a the initial effects of both saltwater and alkali solutions result in an initially higher level of deterioration in tensile strength of FRP system A than deionized water. For the first two months the rate of decrease is 2.4%/month, 7.5%/month, and 10.5%/month as a result of immersion in deionized water, saltwater, and alkali solution, respectively. This is followed by a period of slower decrease between 2 and 6 months followed by an additional decrease over 6 and 18 months for water and saltwater and between 6 and 12 months for the alkali solution. It is interesting that while the overall level of decrease at the end of the 60 month period is 26.4%, 29.7% and 31.7% due to immersion in deionized water, saltwater, and alkali solution, respectively, the corresponding drop in the first 18 months is 19.7%, 21.4% and 26.5% indicating that between 72.1% and 83.6% of the overall drop occurs in the short-term. This is of special interest since most short-term effects are largely reversible on drying although repeated wet-dry cycles can result in matrix microcracking and fiber–matrix debonding which are then irreversible. Despite the large drop in tensile strength, it is interesting to note from Figure 8b that there is very little degradation in tensile modulus with the decreases being 7.3%, 8.2%, and 9.7% due to immersion in deionized water, saltwater, and alkali solution, respectively. As expected from the earlier discussion of the correlation between tensile strength and SBS strength there is a significant drop in SBS strength over the 60-month period of immersion. However, as seen in Figure 8c, the difference between the three solutions is minimal with levels of decrease over the first 6 months being 31.5%, 31.8%, and 34.4%, as a result of immersion in deionized water, saltwater, and alkali solution, respectively, with the corresponding drops over the 60-month period being 43.1%, 44.1% and 43.3%, respectively. While the responses at the end of the period of investigation are asymptotic and within overall scatter, it should be noted that the specimens immersed in the alkaline solution do have a slightly greater level of deterioration. Based on a significantly shorter period of exposure of 20 weeks, Kafodya et al. [69] reported drops in SBS strength of 22.3% and 25.9% due to immersion in water and seawater, respectively, which compare well to the levels attained at the earlier periods of exposure in the current study. In addition, Li et al. [70] reported reductions of 21.52%, 15.65%, and 10.59% in the interlaminar shear strength of CFRP after just 12 weeks of immersion in acidic solution, saline solution, and distilled water, again showing the least effect being due to immersion in water. Given that the extent of deterioration changes within specific time intervals, it is of interest to not just compare levels of decrease but also rates of decrease within the periods so as to better identify zones/stages where mechanisms may be different and/or accelerated. A comparison of rates shows that the rate of decrease for the three solutions—deionized water, saltwater and alkali solution—is 12.7%/month, 9.9%/month, and 11.7%/month over the first two months where immersion in deionized water is the highest, followed by rates of 2.1%/month, 3.7%/month, and 3.6%/month for the next 4 months changing the extent of effect, and then being 0.2%/month from 24 months to 60 months for deionized water, and 0.25%/month for seawater, but a significantly lower rate of decrease of 0.09%/month for alkaline solution resulting in the end level being similar even though the initial decrease due to immersion in alkaline solution was greater. This suggests that long-term degradation is due to fiber–matrix debonding rather than matrix level changes through reactions with different ions in solution.

**Figure 9:**
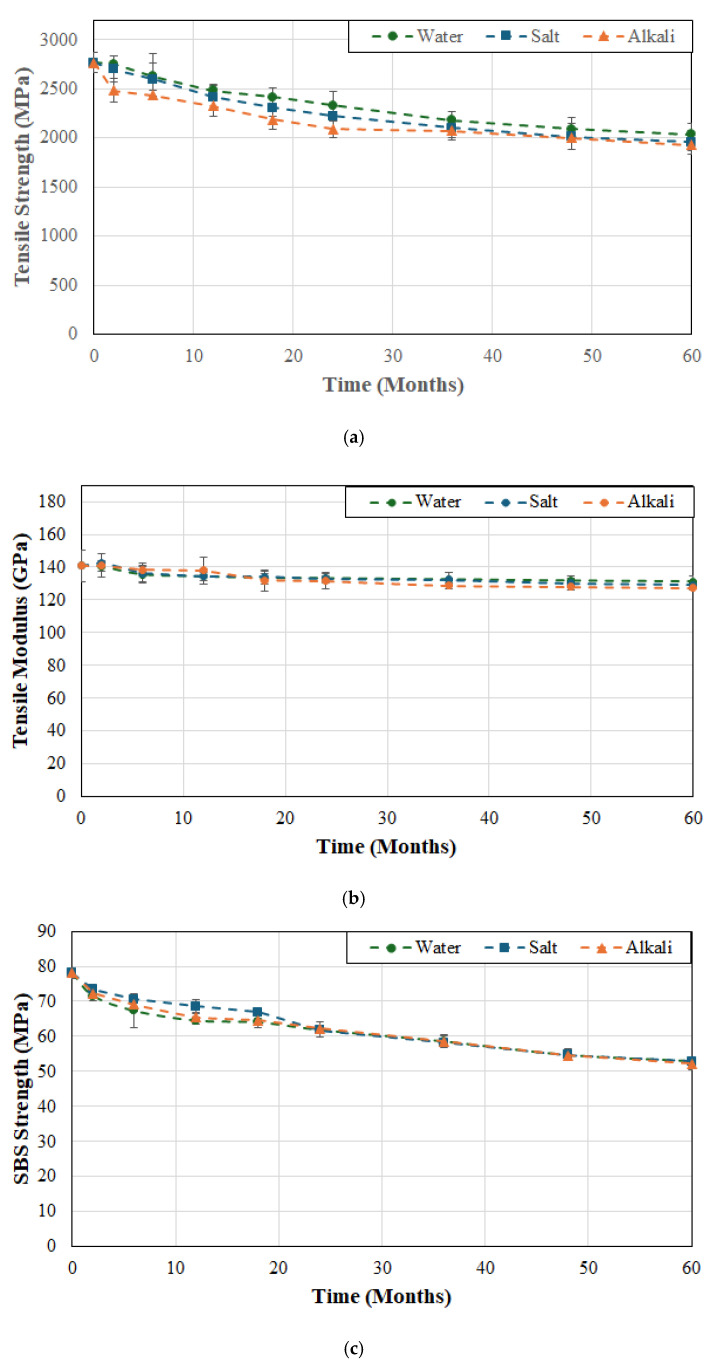
(**a**): Change in tensile strength of FRP system B as a function of solution type and time of immersion. (**b**): Change in tensile modulus of FRP system B as a function of solution type and time of immersion. (**c**): Change in SBS strength of FRP system B as a function of solution type and time of immersion.

As seen from Figure 9a–c, while the response to solutions is more gradual in the case of FRP system B, the overall mechanisms and trends are similar with the effect of immersion in deionized water having the least overall effect and that due to alkaline solution having the most. In this case, however the effect of the three solutions is more similar with tensile strength decreasing over the 60-month period by 26.4%, 29.2% and 30.3% as a result of immersion in deionized water, saltwater, and alkali solution, respectively, suggesting better overall fiber–matrix integrity in the case of FRP system B, which was also seen as a result of elevation in temperatures of immersion in deionized water. The overall trend for the drop in tensile modulus, as seen in Figure 9b, suggests that all three are within scatter bounds almost through the entire 60-month period with the decrease at the end being 7.7%, 8.3%, and 9.6% as a result of immersion in deionized water, saltwater, and alkali solution, respectively, which is roughly equivalent to that of system A, which is not unexpected for a fiber dominated characteristic. The convergence over longer periods of exposure across all three solutions again indicates that while solution-specific mechanisms can cause initial differences in levels of deterioration, interfacial debonding remains the dominant failure mechanism over extended periods of immersion and hence the asymptotic end stage is reached at different rates based on solution type. Uthaman et al. [71] suggested that once the fiber–matrix interface attained a saturation/maximum state further exposure continues at comparable rates across environments. Given the lesser effect on tensile strength, it is not unexpected that the drop in SBS strength would also be less with decreases of 32.1%, 32.2% and 33.1% because of immersion in deionized water, saltwater, and alkali solution, respectively. It is of interest to note that the rate of decrease in the first two months is 4.1%, 2.95% and 3.7% for the three solutions showing that the effect of deionized water is initially greater. In the long-term period from 24 months to 60 months, the averaged rate of decrease is 0.4%/month for both deionized water and saltwater but slightly faster at 0.45%/month for the alkali solution.

**Figure 10:**
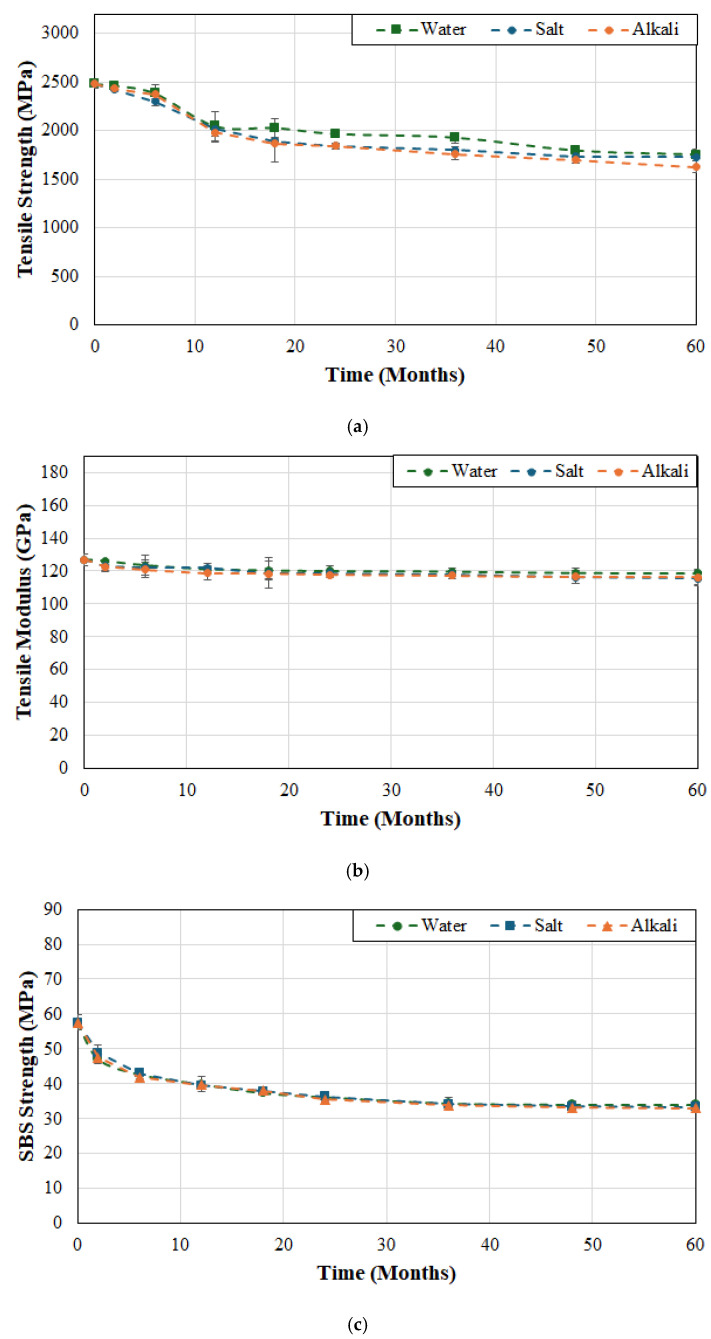
(**a**): Change in tensile strength of FRP system C as a function of solution type and time of immersion. (**b**): Change in tensile modulus of FRP system C as a function of solution type and time of immersion. (**c**): Change in SBS strength of FRP system C as a function of solution type and time of immersion.

Of the three FRP systems, system C indicates the least difference in response to immersion in the three different solutions. As shown in Figure 10a, tensile strength shows a slow decline over the first 6 months with a decrease of 4%, 7.2%, and 4.3% as a result of immersion in deionized water, saltwater, and alkali solution, respectively, with a further drop to levels of 17.6%, 18.7% and 20.2% degradation at the end of 12 months. The overall decrease in tensile strength at the end of 60 months of immersion is noted to be 29.4%, 30.3%, and 34.4%, effectively indicating that about 60% of overall degradation occurs in the first 12 months. As seen in Figure 10b there is very little difference in the trajectory of tensile modulus with time of immersion in the three different solutions with immersion in saltwater having the greatest effect at a 9% decrease and deionized water having the least at 8.5%. The change in SBS strength is much smoother than with the two other systems with the first 6 months still showing a majority of the overall decrease with levels of deterioration of 25.8%, 25.3%, and 27.2% as a result of immersion in deionized water, saltwater, and alkali solution, respectively, as compared to the overall corresponding deterioration of 40.9%, 42%, and 42.8% at the end of the 60-month period of immersion. Over the longer-term period from 24 to 60 months of immersion the averaged rate of decrease is 0.17%/month, 0.22%/month, and 0.2%/month, as a result of immersion in deionized water, saltwater, and alkali solution, respectively, resulting in similar, and the lowest overall, rates of all systems for water and seawater.

**Figure 11:**
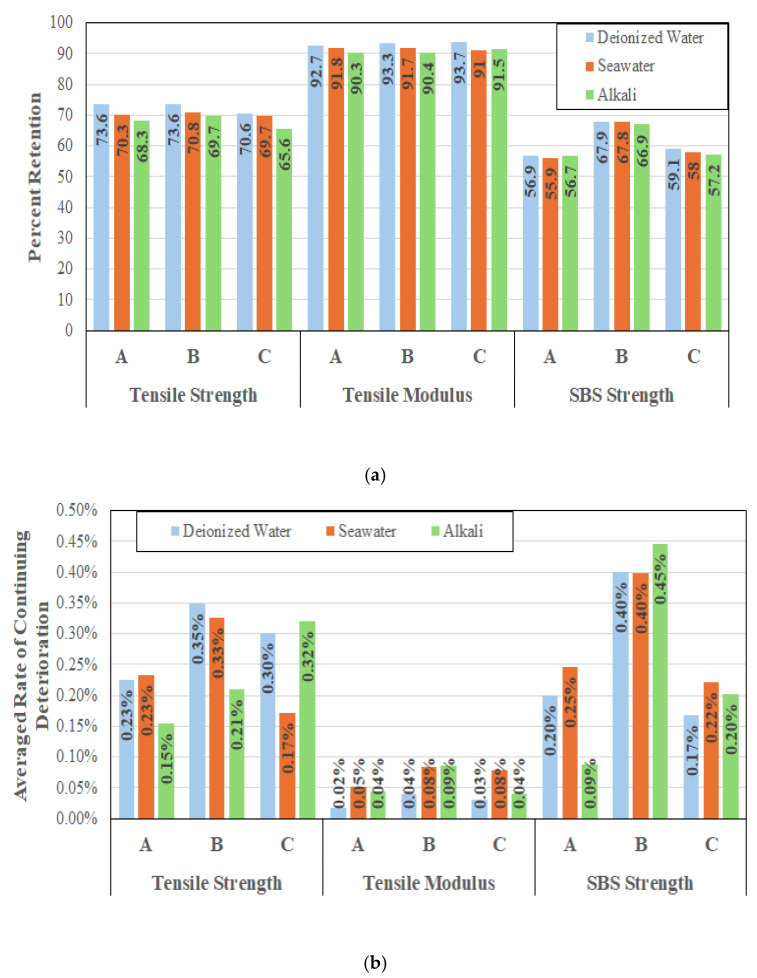
(**a**): Percent retention of performance characteristics at the end of the 60-month period of exposure as a function of solution type. (**b**): Averaged rate of continuing deterioration over the 24–60-month period of immersion as a function of solution type.

Given the interest in long-term characteristics it is of interest to compare responses of the three systems in terms of retention of tensile strength, tensile modulus, and SBS strength at the end of the 60-month period of immersion, as in Figure 11a, and in terms of averaged rates of continued degradation for the three characteristics over the longer-term exposure period of 24 to 60 months, as in Figure 11b.

As can be seen, immersion in alkali (concrete pore water) solution results in the greatest decrease in tensile strength with levels from immersion in deionized water and saltwater being similar albeit with slightly greater deterioration in saltwater. This suggests a level of interaction with the resin resulting in greater hydrolysis and resin deterioration in the presence of alkali ions. The accelerated, early stage, deterioration observed as a result of immersion in saltwater and alkali solution follows the results reported earlier [57,72] which identified ionic diffusion and ion-catalyzed hydrolysis as initiators of early weakening of the fiber–matrix interface. Cabral-Fonseca et al. [31], through flexural tests, also concluded that alkaline solution caused the most severe deterioration. In comparison, modulus retention is extremely high over all three systems and solutions of immersion with very little actual difference overall and even between solutions. The tight range of 90.3–93.7% retention is well within scatter bounds and emphasizes that modulus is a fiber dominated characteristic and with all specimens being unidirectional and processed under tension there is very little, if any, change in linearity and hence in the tensile modulus in the longitudinal direction even with plasticization and fiber–matrix debonding. The largest decrease across all systems is in terms of SBS strength indicating loss in fiber–matrix bond integrity resulting in deterioration in interfacial characteristics. Again, the long-term effects of solution are not significantly different with immersion in the alkaline solution representing the greatest effect and those due to immersion in deionized water the least. While system B shows the best SBS retention, it is noted that the rates of continuing deterioration are also the highest, as shown in Figure 11b, suggesting that over time this aspect may not hold.

It is worth noting that while Figure 11b represents the continuing rates of deterioration averaged over the longer-term exposure period of 24 to 60 months where the deterioration appears to be largely asymptotic, as seen in Figure 8, Figure 9 and Figure 10, the rates continue to decrease over time as would be expected. Table 5 provides a comparison of deterioration rates over a range of longer-term periods for SBS strength of the three systems accruing from immersion in deionized water, seawater, and alkali solution. As can be seen, the rates in the last 12 months (from the 48th month to the 60th month of immersion) are significantly lower than those from the 36-month period of assessment if the asymptotic range of 24 to 60 months were considered, except in the case of system A, where the rate actually more than doubles as a result of immersion in the alkali solution suggesting specific susceptibility to the alkaline ions. The most dramatic change is in system C, where rates decrease significantly in the last 12 months, but even here the drop is less for the alkaline environment.

### 3.4. Prediction of Long-Term Aging

**Figure 12:**
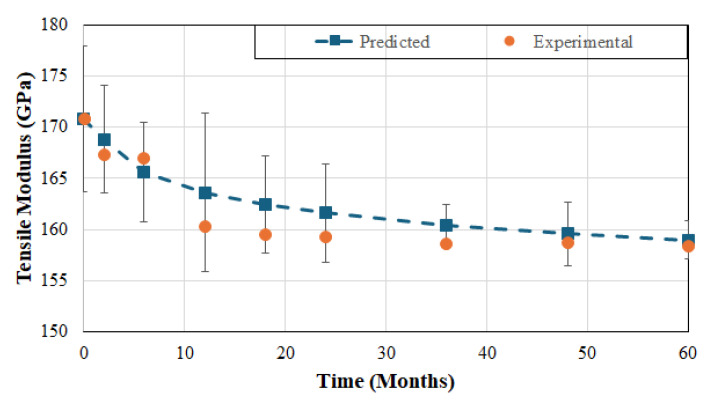
Comparison of Arrhenius-based predictions with experimental data (bars around the experimental averages indicate the maximum and minimum of the set of data to show scatter bound).

To estimate long-term response of the material sets considered in this investigation the Arrhenius model was used assuming that the rate at which degradation occurs, k, follows the form

(4) $$k=exp\left\{\frac{{-E}_{a}}{RT}\right\}$$

where E_a_ is the activation energy, R is the universal gas constant, and T is the exposure temperature in degrees Kelvin. The primary, and gross, approximation and assumption used herein is that the material has a dominant mode of deterioration that does not change with time and temperature but is accelerated by increase in temperature. Notwithstanding the differences seen in the hygrothermal degradation of polymers and polymer matrix composites, the use of the Arrhenius model, especially as related to rates and relative activation energies, has been seen to provide useful information on the long-term durability of materials systems based on limited-term data, and for the development of design thresholds. In the current case, given that the dominant mode of deterioration relates to fiber–matrix interface weakening and debonding, followed by matrix hydrolysis, the assumption can be justified as a first-order approximation. While there are concerns related to the extrapolation of short-term data to very much longer time scales, the existence of 5 years of data in the current case mitigate this to a much larger extent than in previous cases where short-term data, from a few months to a year, were used. It should, however, be cautioned that the results provide a useful means for comparison between materials based on estimation of long-term response, rather than in predicting exact levels of service-life. Following the procedure in Litherland et al. [73] and Proctor et al. [74] and as used for E-glass/vinylester systems by Chin et al. [75] and Karbhari [76], the natural logarithm of time to reach a set of levels of normalized performances versus 1/T can be used to predict service life at a given temperature normally taken as the ambient, or service, temperature. Using data from tests conducted after exposure at the three different temperatures, predictions were made for property retention as a function of time for the 23 °C immersion case. An example of the comparison between experimental data and predictions is shown in Figure 12. As can be seen the predictions are well within the overall scatter levels (shown as maximum and minimum bars around the mean in Figure 12) of the experimental data and compare well with the average levels at longer periods of exposure.

Equations for prediction of long-term performance can then be described by fitting an equation of the type

(5) $$P\left(t\right)=P_{0}\left\{1-A.\;ln\left(t\right)\right\}$$

where P(t) and P_0_ are performance characteristics at time, t, of exposure (in months) and the initial, unexposed, levels, respectively, and A is a degradation rate factor, such that the part within the braces (i.e., { }) represents retention of that characteristic at time, t. Equations for the three FRP systems as related to tensile strength, tensile modulus, and SBS strength as a result of long-term immersion in deionized water are given in Table 6. As can be seen the rate constants change with both FRP systems and characteristic and follow the trends described earlier for deterioration rates.

These results can be extended to the two other immersion conditions of saltwater and alkali solution using procedures of partial safety factors introduced by Karbhari and Abanilla [55] whereby Equation (5) is modified through the use of an environment based partial factor, f_env_, as

(6) $$P\left(t\right)={f_{env}P}_{0}\left\{1-A.\;ln\left(t\right)\right\}$$

where f_env_ is the factor representing the difference in deterioration between deionized water and other immersive environments under the assumption of self-similar damage mechanisms. Using the approach in Karbhari and Abanilla [55] based on attainment of an asymptotic moisture absorption profile after 24 months, factors as listed in Table 7 can be determined for f_env_.

As can be seen from Table 7, the consideration of saltwater or alkaline solution results in a slight decrease in the overall level of performance, with the alkaline environment, in general, having a larger effect which is in line with results reported by Xiong et al. [77]. While the levels of decrease are small, 0–7% for the prefabricated strips, there is also significant similarity in overall response between the three systems, suggesting that all three would behave in a similar fashion over an extended period of immersion, as was indicated by a comparison of rates of deterioration over the longer term in an earlier section.

It should be emphasized that all these predictions are expected to reflect an aggressive set of exposures since they are based on full immersion over the entire period under consideration, rather than the more commonly seen conditions of humidity and/or periodic immersion. Further, the data are developed on specimens that have not been coated with protective coatings such as gel coats that are usually applied in an offshore/marine or aqueous environment to further reduce moisture uptake. Nonetheless, they present a useful means of estimating differences in response over extended periods of time between competing systems. While the characteristic and guaranteed values provide levels of design thresholds, they do not account for effects of environmental exposure which are considered through the addition of environmental reduction factors such as in ACI-440 [30] wherein design levels are determined through the product of an environmental reduction factor, C_E_, and the guaranteed level. The design ultimate strength, f_fu_, for example, is determined as

(7) $$f_{fu}=C_{E}f_{fu}^{*}$$

where C_E_ = 0.85 for carbon fiber reinforced composites subject to exterior exposure and $f_{fu}^{*}$ is the guaranteed tensile strength. fiB [52] defines the design tensile strength as

(8) $$f_{fud}=\frac{n_{f}f_{fuk}}{\gamma_{f}}$$

where f_fuk_ is the characteristic tensile strength (defined as the average minus 1.645 times the standard deviation), n_f_ is a reduction factor for relevant exposure in accordance with ISO 10406 [78] taken as 0.7, and g_f_ for carbon fiber reinforced polymer strips is 1.3 at the maximum. Applying the format used for tensile strength in Equations (7) and (8) to the tensile modulus and SBS strength enables the determination of design thresholds for each, and these can then be used in conjunction with equations in Table 6 to attain times to reach the thresholds which are reported in Table 8.

As can be seen, the ACI-440 levels for tensile strength and SBS strength are attained at very short periods of exposure and well within the experimental period of investigation. In comparison, the levels prescribed by fiB for tensile strength are attained after over 50 years of immersion while those for SBS strength are still within the 5-year period for systems A and C. In all cases, the tensile modulus does not show any indicator of attaining the threshold till significantly past a 100-year estimate. Of all systems, FRP system B is seen to have the best overall response. It should, however, be emphasized again that conditions of full immersion without protective coatings is not expected in field applications and hence the actual design life can be expected to be significantly higher.

Given the low values of design life using the environmental reduction factor specified by ACI-440 [30], it is of interest to determine the value of the factor that would be needed to be applied to attain a specified design life. This can be determined through restructuring of Equation (5) such that

(9) $$C_{E}=\frac{Average\;Value}{Guaranteed\;Value}\;\left\{1-A.ln\left(t_{T}\right)\right\}$$

where average and guaranteed refer to the average and guaranteed values of the mechanical performance characteristic, respectively, A is the degradation rate factor as listed in Table 6, and t_T_ is the target design life in months (e.g., a target design life of 10 years would be t_T_ = 10 × 12 = 120 months). Values for the environmental reduction factor based on immersion in deionized water at 23 °C are listed in Table 9.

As would be expected, all values decrease with the increase in intended design life, and with the exception of tensile modulus where the ACI-440 value of 0.85 is more than adequate, the values are lower than the threshold of 0.85. In particular the SBS strength levels are extremely different, which is not surprising considering that interfacial/shear failure within the composite is not considered by the standard since the traditionally required characteristics are those of unidirectional strength, modulus, and strain. However, shear response is a critical aspect in unidirectional composites and is a mechanism that could lead to catastrophic, albeit rare, failure and hence should be studied in greater depth. It is also noted that the values of C_E_ do not drop substantially as design life is increased from 50 to 100 years because of the asymptotic nature of the predictive equation formulated based on the Arrhenius concept of single dominant mechanism and self-similar response over the extrapolated period of time. The reader is, however, cautioned that, as stated earlier, this is a gross approximation and the accuracy of extrapolations to such long periods of time based even on 5 years of experimental data cannot be validated, nor guaranteed. In general, however, a factor of 0.5 would suffice for all three systems, which is not significantly different from the value of 0.538 obtained from the ratio of $\frac{n_{f}}{\gamma_{f}}$ used in the fiB standard.

## 4. Summary and Conclusions

Although the method of external bonding of CFRP strips is increasingly being used as a means of strengthening deteriorating and understrength structural concrete components, there is still a lack of a comprehensive database related to the characterization of different commercially available systems with a view to developing a set of material specifications for a standardized product similar to that available with traditional materials, and of long-term data that could be used to assess durability and predict service-life. Most laboratory tests range from a few months to perhaps 1–2 years, which does not provide a long enough period for reasonable extrapolation of data. The current study provides a unique set of data on three different prefabricated CFRP composite systems that were tested in tension and short beam shear after immersion in water, seawater, and alkaline solution representative of concrete pore water, over a period of 5 years. Exposure to deionized water was conducted at three temperature levels, significantly lower than the glass transition temperature, to enable the use of Arrhenius principles for prediction of long-term response. Results were then compared to the factors used by different codes to assess their appropriateness for use in design. The following primary conclusions can be made:

- The three systems not only vary in thickness, although they were designed for the same application, but also in fiber volume fraction. Data sets for unexposed specimens collected and tested over the 5-year period provide a reasonable set for characterizing distributions of the tensile strength and modulus and SBS strength, and it is shown that the three systems cannot be pooled together and show very different distribution responseThe level of deterioration increases with temperature of immersion in deionized water with the least effect being seen on tensile modulus and the greatest in terms of SBS strength.
- Tensile modulus is affected the least over the 5-year period of exposure with the levels of deterioration being similar across all three systems ranging from 7.3–11.9%.
- Tensile strength is seen, for the most part to decrease significantly over the first 6 months of exposure followed by a decreased rate of deterioration. Immersion in deionized water at 60 °C results in the highest level of degradation between 33.4% (System B) and 38.2% (System C), with levels of deterioration being the highest for the alkaline solution and the least in deionized water suggesting the effect of ions in solution on the interface and in breaking the weak hydrogen bonds in the polymer chain. SBS strength is seen to deteriorate dramatically in the first 12 months with a reduced level thereafter, but in all cases both to a greater extent and at a faster rate than the deterioration in tensile strength emphasizing the effect of deterioration at the fiber-matrix and interfacial level which is obscured to an extent by longitudinal tensile tests on unidirectional materials. Predictions of long-term durability derived using Arrhenius principles compare well with experimental data over the 5-year period of testing. However, the extent of degradation shown both by tests and through predictions suggest that the factors used for environmental exposure in determination of design thresholds are non-conservative for tensile strength, and if extended to SBS strength for that as well. While current design guidelines do not consider SBS strength or other interfacial/intralaminar characteristics, results show that these should be considered in addition to the traditional sets of tests to provide better data regarding deterioration and to provide warning of how a FRP system could fail in modes of loading other than pure tension in the fiber direction.

The tests provide a comprehensive evaluation of characteristics through longer-term laboratory testing. It is however, emphasized that these results do not directly relate to complex, and changing, field exposures for which correlations are still being pursued by investigators globally. Further since tests were conducted under conditions of full immersion without sealing of edges and/or use of surface/gel coatings the laboratory tests should be considered to represent harsher conditions than might be seen in the field. Notwithstanding these the tests do show trends that mimic the data from limited field specimens removed from existing structures raising a level of caution regarding assumptions related to long-term durability. Further testing, especially over extended periods of exposure both in the field and using multiple environmental conditions in the laboratory, is needed as is significantly more analysis for determination of product specifications and methods of determining long-term durability.

## Figures and Tables

**Table 1:** Details of FRP strip systems.

System	Fiber Volume Fraction (%)	Density (g/cm^3^)	Nominal Thickness (mm)	Glass Transition Temperature (°C)
A	69	1.59	1.3	157.9
B	61	1.55	1.3	145.6
C	62	1.53	1.9	140.1

**Table 2:** Unexposed “Baseline” characteristics (numbers in [ ] are standard deviations).

System	Tensile Strength (MPa)	Tensile Modulus (GPa)	SBS Strength (MPa)
A	2829.46 [63.11]	170.81 [7.13]	62.72 [3.17]
B	2767.58 [103.77]	140.79 [9.84]	78.04 [1.48]
C	2481.65 [51.38]	126.77 [3.37]	57.63 [2.04]

**Table 3:** Summary of Weibull parameters.

Characteristic.	FRP System	Shape Parameter (a)	Scale Parameter (b)
Tensile Strength	A	38.096	2821.013
	B	30.950	2786.644
	C	54.926	2512.205
Tensile Modulus	A	26.265	173.639
	B	27.709	143.832
	C	89.763	127.381
Short Beam Shear Strength	A	27.545	62.382
	B	43.295	79.311
	C	22.210	58.789

**Table 4:** Results of tests for normality and Weibull fit.

Characteristic	FRP System	Shapiro-Wilk Test		Kolmogorov-Smirnov Test				
				K-S Normal		K-S Weibull		Best Fit
		*p*	Normality	KS Stat	*p*	KS Stat	*p*	
Tensile Strength	A	0.011	No	0.111	0.593	0.08	0.912	Weibull
	B	0.003	No	0.163	0.164	0.102	0.704	Weibull
	C	0.104	Yes	0.122	0.478	0.167	0.147	Normal
Tensile Modulus	A	0.610	Yes	0.085	0.877	0.122	0.476	Normal
	B	0.628	Yes	0.085	0.874	0.12	0.494	Normal
	C	0.146	Yes	0.079	0.918	0.138	0.331	Normal
SBS Strength	A	0.352	Yes	0.106	0.646	0.15	0.235	Normal
	B	0.891	Yes	0.061	0.99	0.114	0.559	Normal
	C	0.151	Yes	0.128	0.439	0.1	0.719	Weibull

**Table 5:** Deterioration rates (change in SBS/month) for SBS strength.

System	Time Period	Immersion Solution		
		Deionized Water	Seawater	Alkali
A	24–60 months	−0.200%	−0.246%	−0.087%
	36–60 months	−0.228%	−0.215%	−0.098%
	48–60 months	−0.114%	−0.147%	−0.178%
B	24–60 months	−0.401%	−0.399%	−0.447%
	36–60 months	−0.401%	−0.380%	−0.450%
	48–60 months	−0.268%	−0.283%	−0.355%
C	24–60 months	−0.168%	−0.221%	−0.202%
	36–60 months	−0.050%	−0.084%	−0.105%
	48–60 months	−0.012%	−0.011%	−0.065%

**Table 6:** Predictive equations for performance characteristics of the FRP systems.

System	Tensile Strength	Tensile Modulus	SBS Strength
A	2829.46{1 − 0.0717ln(x)}	170.81{1 − 0.0169ln(x)}	62.72{1 − 0.1387ln(x)}
B	2767.58{1 − 0.0603ln(x)}	140.79{1 − 0.0111ln(x)}	78.04{1 − 0.0949ln(x)}
C	2481.65{1 − 0.0746ln(x)}	126.77{1 − 0.0155ln(x)}	57.63{1 − 0.1300ln(x)}

**Table 7:** Environment Based Partial Factor for Prefabricated Strips.

System	Tensile Strength		Tensile Modulus		SBS Strength	
	Saltwater	Alkali	Saltwater	Alkali	Saltwater	Alkali
A	0.95	0.93	0.95	0.93	0.99	0.98
B	0.96	0.94	0.99	0.97	1	1
C	0.96	0.93	0.98	0.98	0.99	0.97

**Table 8:** Estimated time to reach design thresholds after immersion in deionized water at 23 °C.

Threshold	Standard	Time (Years) to Reach Threshold		
		A	B	C
Tensile Strength	ACI-440	1.5	4.9	1.3
	fiB	68.6	304.9	51.8
Tensile Modulus	ACI-440	>100	>100	>100
	fiB	>100	>100	>100
SBS Strength	ACI-440	0.6	0.7	0.5
	fiB	3.2	12.9	3.7

**Table 9:** Environmental reduction factors based on desired design life.

Design Life (Years)	Tensile Strength			Tensile Modulus *			SBS Strength		
	A	B	C	A	B	C	A	B	C
10	0.70	0.80	0.69	1.05	1.19	1.01	0.40	0.58	0.42
25	0.63	0.74	0.61	1.03	1.19	0.99	0.25	0.49	0.29
50	0.58	0.69	0.56	1.02	1.18	0.98	0.13	0.42	0.19
100	0.53	0.65	0.50	1.01	1.17	0.97	0.02	0.35	0.09

* The ACI-440 stated value of 0.85 is conservative and can be used here.

## Data Availability

The original contributions presented in this study are included in the article. Further enquiries can be directed to the corresponding author.
